# Reliability and Quality of YouTube Contents Pertaining to Pancreatic Cancer

**DOI:** 10.7759/cureus.14085

**Published:** 2021-03-24

**Authors:** Guner Cakmak, Baris Mantoglu

**Affiliations:** 1 Department of General Surgery, Sakarya Training and Research Hospital, Sakarya, TUR

**Keywords:** pancreatic cancer, youtube, discern, gqs, vpi

## Abstract

Objective: In our study, we aim to evaluate in terms of patients the quality and reliability of the most relevant and most-watched videos uploaded on YouTube about pancreatic cancer.

Method: Before starting the study, YouTube^TM^ search terms were determined by consensus by two General Surgeons. Then, on 10/10/2020, the terms such as “pancreatic cancer”, “diagnosis of pancreatic cancer” and “treatment of pancreatic cancer” were entered separately in the search bar of YouTube, “relevance” was selected among the filtering options and the most viewed videos were listed. The videos were evaluated with the Global Quality Scale (GQS), the DISCERN scoring system (Quality Criteria for Consumer Health Information, http://www.discern.org.uk), and video power index.

Results: Among the 50 videos analysed, 19 videos were uploaded by hospital channels, 17 videos by health channels, seven videos by patients, four videos by blog channels, and three videos by doctors. The mean GQS score of the first researcher was 3.24 ± 0.99 and the mean GQS score of the second researcher was 3.18 ± 0.88 with a significantly high agreement between them (r= 0.628). The mean DISCERN score of the first researcher was 3.48 ± 0.77 and the mean DISCERN score of the second researcher was 3.46 ± 1.09 with a significantly high agreement between them (r= 0.814).

Conclusion: In our study, the majority of the videos were found to be of moderate quality. Healthcare professionals should be encouraged to upload more videos with useful content. However, we think that the uploaded videos should definitely go through a professional peer-review process before they are published.

## Introduction

With more than 50,000 new cases and over 90% mortality reported each year in the United States, pancreatic cancer (PC) is one of the deadliest malignancies [1,2]. PC can be difficult to diagnose. Because of its non-specific and atypical symptoms, PC is often diagnosed at advanced stages [3-6]. Survival varies depending on the stage at which the PC is diagnosed. If the tumor has metastasized to distant parts of the body, survival rate is only 3% [7].

Today, patients and their relatives are trying to obtain information from various sources on PC, which is difficult to detect and treat and has a poor prognosis. The Health Information National Trends Survey revealed that nearly half of people searching for cancer information turn to the internet first, rather than seeking medical advice [8,9].

Today, the internet and, in particular, platforms such as YouTube (www.youtube.com) where visual content is abundantly available, are the most important and widely used source of information for people of all ages worldwide. According to 2020 statistics, there are two billion YouTube users worldwide; one billion hours of videos are watched on YouTube every day and 400 hours of video are uploaded to YouTube every minute worldwide [10]. Some studies have found that patients report that the information they obtain over the internet is at least as reliable as that given to them by doctors, or even more so [11].

In the past, various studies have been carried out to investigate the quality and reliability of video contents related to health issues uploaded to YouTube and whether they are beneficial for patients [12,13]. However, we did not find any study on PC in our literature search.

Therefore, in this study, we aim to evaluate in terms of patients the quality and reliability of the most relevant and most-watched videos about PC uploaded to YouTube.

## Materials and methods

Data collection

Before the study began, YouTube search terms were determined by consensus by two general surgeons. Then, on 10/10/2020, the terms “pancreatic cancer,” “diagnosis of pancreatic cancer,” and “treatment of pancreatic cancer” were entered separately in the search bar of YouTube. “Relevance” was selected among the filtering options and the most viewed videos were listed. After excluding news, repeated videos, videos in languages other than English, and those that were less than 120 seconds or contained advertising content, the top 50 most relevant and most-watched videos were recorded. In addition, the qualification of the uploader, video content, video length (seconds), upload date, time elapsed since upload, number of views per day, number of likes and dislikes, number of comments, and video power index (VPI) were also recorded. The quality of the videos was evaluated by VPI values calculated according to the formula: VPI = like count/(like count + dislike count) x 100 [12,13]. 

Data analysis

The collected data were separately evaluated and scored by two general surgeons with 22 and 12 years experience on panceratic cancer, respectively. The videos were evaluated at the same time, but in different locations, in order to prevent bias during the evaluation. We used the Global Quality Scale (GQS) [14,15], which has been used in many studies in the literature, in order to determine the quality of the videos. The five categories of the GQS are: 1- Poor quality, poor flow of the content, most information missing, not at all useful for patients; 2- Generally poor quality and poor flow, some information listed, but many important topics missing, of very limited use to patients; 3- Moderate quality, suboptimal flow, some important information is adequately discussed, but other information is poorly discussed, somewhat useful for patients; 4- Good quality and generally good flow, most of the relevant information is listed, but some topics not covered, useful for patients; and 5- Excellent quality and excellent flow, very useful for patients.

In the 5-point scale of GQS, 1 point is associated with poorly useful, 2 points with poorly useful (limited use), 3 points with somewhat useful, 4 points with useful, and 5 points with highly useful. Each question listed below that is answered with a “yes” corresponds to 1 point and those answered with “no” receive 0 points. A maximum of 5 points can be obtained. High scores indicate the reliability of the video content. The 5-item questionnaire of the DISCERN scale (Quality Criteria for Consumer Health Information, http://www.discern.org.uk) is: 1- Are the aims clear? 2- Are reliable information sources used (e.g. physicians, health channels etc.)? 3- Is the information balanced and unbiased? 4- Are additional sources listed for patients? 5- Are areas of uncertainty stated?

As a result of the researchers' evaluations, 1 or 2 points obtained from the GQS and DISCERN scales indicate that the video content is misleading, 3 points indicate that the video content is of moderate quality and reliability, and 4 or 5 points indicate that the video content is useful for patients.

Ethics statement

Since our study did not include human or animal subjects and only analyzed videos on the YouTube platform, ethical approval was waived.

Statistical analysis

Data were analyzed using Statistical Package for Social Sciences (SPSS) version 23 (IBM Corp., Armonk, NY, USA). During the evaluation of the study data, frequencies (number, percentage) of categorical variables and descriptive statistics (mean, standard deviation, median, minimum, and maximum) of numerical variables were given. Normality assumptions of numerical variables were examined with the Shapiro Wilk normality test and it was found that they were not normally distributed. Therefore, nonparametric statistical methods were used in the study. The relationship between two independent numerical variables was interpreted with Spearman's Rho correlation coefficient. Differences between two independent groups were examined by Mann-Whitney U analysis. The correlation between two rates was examined by Spearman’s correlation analysis and the agreement between them was evaluated using alpha coefficients. P<0.05 values were considered statistically significant.

## Results

A total of 50 of the most relevant and watched videos about PC were included in the scope of the study. The average video length, total number of views, time elapsed since the upload of the video, daily number of views, average comments, likes, dislikes, and VPI values are given in Table 1. 

**Table 1:** Descriptive statistics of video features VPI: video power index

	Mean ± SD	Median	Min	Max
Video length (sec)	497.1 ± 369.1	440.00	137	1440
Number of views	248.810 ± 741.814	72.100	3.735	5.251.574
Time elapsed since the upload of the video (days)	1671.9 ± 902.70	1854	77	3.945
Number of views (daily)	279.77 ± 1.123,70	54.99	3.3	7.981
Comments	459 ± 2.184,3	93	0	15.541
Likes	4.012 ± 213.92	394	14	152
Dislikes	103.62 ± 290.10	25	1	2.000
VPI (%)	92.081 ± 8.024	96.721	56.858	99.17

Among the 50 videos analyzed, 19 videos were uploaded by hospital channels, 17 videos by health channels, seven videos by patients, four videos by blog channels, and three videos by doctors. The distribution of the videos by the qualifications of the uploaders and the general features of the videos are given in Table 2.

**Table 2:** General features of the videos

(n=50)	Number	Percentage
Image Type		
Real	43	86
Animated	7	14
Qualifications of Video Uploaders		
Health channel	19	38
Hospital channel	17	34
Patient	7	14
Blog channel	4	8
Doctor	3	6
Video Content		
Patient experience	20	40
General information	18	36
Surgical technique	8	16
Diagnosis	3	6
Treatment	1	2

When VPI values were separately examined according to the qualifications of the uploaders, we found that the average VPI value of videos uploaded by hospital channels was 90.87 ± 4.59, the average VPI of videos uploaded by hospital channels was 90.99 ± 7.98, the average VPI of videos uploaded by patients was 94.77 ± 2.48, the average VPI of videos uploaded by doctors was 91.79 ± 1.55, and the average VPI of videos uploaded by blog channels was 97.89 ± 0.74.

The distribution of the videos by the video content and the qualifications of uploaders are given in Table 3.

**Table 3:** Distribution by the video content and the qualifications of uploaders

	Health Channel	Hospital Channel	Patient	Blog Channel	Doctor	TOTAL
Patient Experience	8	4	7	1	0	20
General Information	7	7	0	2	2	18
Surgical Technique	2	5	0	0	1	8
Diagnosis	2	0	0	1	0	3
Treatment	0	1	0	0	0	1
TOTAL	19	17	7	4	3	50

The number of comments, likes, and dislikes and the VPI values according to the content of the videos are given in Table 4.

**Table 4:** Number of comments, likes, and dislikes and VPI values by the content of the videos VPI: video power index

Content	Number	Comments	Likes	Dislikes	VPI (%)
Patient experience	20	980 ± 2.227	8.350 ± 21.818	162 ± 295	93.67 ± 8.41
General information	18	125 ± 194	1.255 ± 1.180	88 ± 8	91.16 ± 7.0
Surgical technique	8	50 ± 183	717 ± 1.249	87 ± 8	87.34 ± 8.0
Diagnosis	3	202 ± 189	4.800 ± 1.085	38 ± 49	98.35 ± 7.0

Ten (20%) of the 50 videos included in the study were animated and the other 40 (80%) videos were real images. It was determined that the average number of comments for animated videos was 98 ± 185, the average number of likes was 1.866 ± 1.288 and the average number of dislikes was 100 ± 98. In addition, the average VPI of animated videos was found to be 95.19 ± 7.88. It was determined that the average number of comments on the real images was 549 ± 2.162, the number of likes was 4.548 ± 21.177, the number of dislikes was 104.3 ± 290, and the average VPI value was 91.30 ± 7.95.

The number of comments, likes, and dislikes according to the qualifications of the video uploaders is given in Table 5.

**Table 5:** The number of comments, likes, and dislikes by the qualifications of the video uploaders

	Number of Videos	Comments	Likes	Dislikes
Health channel	19	2.140	17.968	1.596
Hospital channel	17	1.106	6.236	441
Patient	7	17.783	157.061	2.281
Blog channel	4	1.399	11.956	273
Doctor	3	525	7.364	586

When Table 5 is examined, it is determined that only seven of the 50 videos were uploaded by patients, but the highest rate of comments and likes is recorded in these videos. The most-watched video in our study was uploaded by a patient in 2019, and this video was viewed 5,251,574 times. The least viewed video was uploaded by a hospital channel in 2020 and was viewed 3,735 times.

The mean DISCERN score given by the two researchers to the videos analyzed was 3.47 ± 0.99 (min-max: 1-5), and we found that the reliability of the videos was of moderate quality. The mean DISCERN score given by the first researcher to the videos was 3.48 ± 0.77 (min-max: 2-5), and the mean DISCERN score of the second researcher was 3.46 ± 1.09 (min-max: 1-5). There was a high inter-rater reliability in terms of the DISCERN scores (r=0.814).

The mean GQS score given by the researchers to the videos was 3.21 ± 0.94 (min-max: 1-5). The mean GQS score of the first researcher was 3.24 ± 0.99 and that of the second researcher was 3.18 ± 0.88 (min-max: 1-5). There was a high inter-rater reliability in terms of the GQS scores (r=0.628). The distribution of DISCERN and GQS scores by the qualifications of the uploaders is given in Table 6. 

**Table 6:** Mean DISCERN and GQS scores of the first and second researchers GQS: Global Quality Scale, DISCERN: Quality Criteria for Consumer Health Information

	DISCERN 1	DISCERN 2	GQS 1	GQS 2
Health channel	3.78 ± 0.86	3.89 ± 1.07	3.68 ± 1.01	3.47 ± 0.90
Hospital channel	3.76 ± 0.81	3.70 ± 1.10	3.52 ± 1.03	3.47 ± 0.90
Patient	2.71 ± 0.93	2.70 ± 1.11	2.14 ± 1.02	2.42 ± 0.82
Blog channel	1.75 ± 1.48	1.50 ± 1.23	1.75 ± 1.28	1.55 ± 1.16
Doctor	4.33 ± 0.91	4.00 ± 0.66	4.00 ± 1.1	4.12 ± 0.81

The mean DISCERN score of the animated videos was 3.8 ± 1.13 and the mean GQS score was 3.3 ± 1.11. The mean DISCERN score of the real image videos was 3.41 ± 1.70 and the mean GQS score was 3.25 ± 1.04. According to this, it was determined that the quality and reliability of videos with both real and animated content were moderate.

Seven (14%) of the 50 videos contain misleading information, and we found that these videos were uploaded by patients and blog channels. These videos were viewed more than 6 million times. The oldest video was uploaded in 2014 and the newest one in 2019.

Eighteen (36%) of the videos contain useful information. Two of these were uploaded by doctors, and 16 by health and hospital channels.

It was found that the remaining 25 (50%) videos were of moderate quality and almost all were uploaded by health and hospital channels.

## Discussion

Studies have reported that eight out of 10 internet users search the internet for their health information [16]. YouTube is the visual media platform that patients and their relatives use the most to get information about their health. YouTube content can provide educational and useful information. However, many studies have shown that health-related videos can contain misleading and harmful information when not uploaded by healthcare professionals [12,13]. For this reason, the number of studies aiming to assess the quality and reliability of YouTube content on various health issues is increasing day by day in the literature. In a study conducted in 2018, it was reported that there were nearly 2,000 studies in which YouTube videos with health content were examined [17]. Studies have been conducted on glioblastoma, social media addiction, human papillomavirus (HPV) vaccines, premature ejaculation, retinopathy, diabetes mellitus, and schizophrenia, and in almost every field of medicine [18]. Although there are studies that have analyzed YouTube videos on many types of cancers [12,19,20], no study has been found on PC.

It was determined that 12 of the 36 videos uploaded by health and hospital channels are patient experience videos. It was found that the mean DISCERN score of these videos was 3.75 and the mean GQS score was 3.5. These videos with a moderate quality score were found to have post-surgical survival content. They only provide information about short-term results, and their uploaders are partially profit-oriented organizations. In Yurdaisik’s study evaluating breast cancer videos, videos uploaded by healthcare professionals were found to be of medium quality [12]. Previous studies have reported that the quality of these videos is poor or moderate [12,13]. Accordingly, it is concluded by researchers that health-related videos should only be uploaded by healthcare professionals. However, the rate of videos uploaded directly by doctors in our study is very low (6%), as in previous studies. Although the contents of videos uploaded by health channels and hospital channels are mostly presented by doctors, they are generally promotional corporate videos and do not reflect the doctors’ own views as healthcare professionals. There could be various reasons for the low rate of videos uploaded by doctors to YouTube. In recent years, the use of social media with the evolution of smartphones has surpassed video platforms such as YouTube. Patients now follow doctors on social media about their own health problems, benefit from one-to-one sharing, and can even participate interactively by watching their live broadcasts. In these broadcasts, patients can directly ask the questions they are curious about and receive their answers in a descriptive way, benefiting the other participants as well. It can be said that this way of expressing their profession is more attractive to doctors than uploading videos to YouTube in terms of both self-improvement and patient guidance. Another reason that doctors refrain from uploading videos to YouTube is the fear of infringing copyright and privacy [21]. While the responsibility for the contents uploaded by corporate health channels belongs to the relevant institution, the responsibility for the content of videos directly uploaded by doctors belongs to the doctors. This suggests that doctors hesitate to upload videos for professional reasons and, for this reason, the proportion of such videos is low. However, it is not a realistic expectation that every video uploaded by doctors is useful and not misleading. In this regard, it is suggested that videos uploaded to platforms such as YouTube are only published after they are audited by a committee of healthcare professionals to confirm their scientific validity and ensure that they are not misleading. Considering the sensitivity of cancer patients and their relatives, such videos are important. Therefore, although professionals should be encouraged to upload videos about health, we think that these videos should go through a peer review process.

Only seven of the 50 videos analyzed were uploaded by patients and all of these videos contain the experiences of the patients. These seven videos have been viewed more than 6 million times in total. The most viewed video has the most comments and the most likes, with more than 5 million views. Videos uploaded by patients have an average daily view rate of 1.201. Five of these seven videos contain misleading information, and two of them are of moderate quality. In studies conducted on similar topics in the literature, it has been reported that the videos uploaded by patients are viewed more and get more likes [13,22-24]. Considering that the diagnosis of PC is usually made at late stages and the surgical chances of these patients are very low, we think that patients and their relatives are in search of different options.

Eight of the videos analyzed have content on surgical technique. These videos are of moderate quality and usefulness and have been viewed 1,134,410 times in total. The VPI values of these videos uploaded by healthcare professionals have been found to be lower than those uploaded by patients and blog channels.

Ten of the videos analyzed are animated videos, and these videos had a lower number of likes than the videos containing real images. Due to the fact that anatomical information is given in animated videos and medical terms are used extensively, we think that these videos are not understood and liked by the audience. However, the VPI values of the animation videos were found to be higher than the VPI values of the videos containing real images.

Fifty videos included in our study have been viewed 12,440,505 times, with an average daily view rate of 280. However, different rates were reported in previous studies where YouTube contents were assessed. In the study conducted by Kunze et al., 50 videos were analyzed and the total number of views was reported as 14,141,285 [25]. However, in the study conducted by Champan et al., 202 videos were analyzed and the total number of views was reported as 2,518,512 [26].

In this study, where we analyzed videos about PC, we found that the videos are generally of moderate quality in terms of content. We included in the scope of the study the most viewed videos after filtering by relevance which appeared when we typed our pancreas-related keywords into the YouTube search bar. In addition, we also included in our study the first videos that appeared when we entered our keywords.

Limitations

This study includes considerable limitations on the data examined. First, we could not fully compare the findings of our study with previous studies conducted in this field. However, since there is no other study similar to ours, we compared our data with YouTube studies in different fields. We evaluated all the most-watched videos related to PC, although such videos were rare. The fact that we analyzed only 50 videos in this study can be seen as a limitation. Finally, since there is no other study in the literature investigating the quality and reliability of YouTube contents on PC, we could not compare our results regarding PC videos. 

## Conclusions

In our study, the top 50 most relevant and most viewed videos on YouTube on PC, a cancer type that is difficult to treat, has a poor prognosis, and a low survival rate, were analyzed. According to our findings, the rate of videos uploaded directly by doctors is very low. Although the DISCERN and GQS scores of these videos are higher than the scores of videos uploaded by non-healthcare professionals, the number of views, comments, and likes of videos uploaded by patients is higher. In our study, the majority of the videos were found to be of moderate quality. Healthcare professionals should be encouraged to upload more videos with useful content. However, we think that the uploaded videos should go through a professional peer-review process before they are published.

## References

[1] Siegel RL, Miller KD, Jemal A (2018). Cancer statistics. CA Cancer J Clin.

[2] Rawla P, Sunkara T, Gaduputi V (2019). Epidemiology of pancreatic cancer: global trends, etiology and risk factors. World J Oncol.

[3] Chang JC, Kundranda M (2017). Novel diagnostic and predictive biomarkers in pancreatic adenocarcinoma. Int J Mol Sci.

[4] Güngör C, Hofmann BT, Wolters-Eisfeld G, Bockhorn M (2014). Pancreatic cancer. Br J Pharmacol.

[5] Mohammed S, Van Buren G, Fisher WE (2014). Pancreatic cancer: advances in treatment. World J Gastroenterol.

[6] Zhang QH, Ni QX, Coordination Group of The Committee on Pancreatic Cancer (2004). Clinical analysis of 2340 cases of pancreatic cancer [Article in Chinese]. Zhonghua Yi Xue Za Zhi.

[7] (2020). Pancreatic Cancer Survival Rates: What Do They Really Mean?. https://cancercommons.org/latest-insights/pancreatic-cancer-survival-rates-what-do-they-really-mean/.

[8] Ho M, Stothers L, Lazare D, Tsang B, Macnab A (2015). Evaluation of educational content of YouTube videos relating to neurogenic bladder and intermittent catheterization. Can Urol Assoc J.

[9] Hesse BW, Nelson DE, Kreps GL, Croyle RT, Arora NK, Rimer BK, Viswanath K (2005). Trust and sources of health information: the impact of the Internet and its implications for health care providers: findings from the first Health Information National Trends Survey. Arch Intern Med.

[10] (2021). 10 YouTube Stats Every Marketer Should Know in 2021. https://www.oberlo.com/blog/youtube-statistics.

[11] Kwakernaak J, Eekhof JA, De Waal MW, Barenbrug EA, Chavannes NH (2019). Patients' use of the internet to find reliable medical information about minor ailments: vignette-based experimental study. J Med Internet Res.

[12] Yurdaisik I (2020). Analysis of the most viewed first 50 videos on YouTube about breast cancer. Biomed Res Int.

[13] Kuru T, Erken HY (2020). Evaluation of the quality and reliability of YouTube videos on rotator cuff tears. Cureus.

[14] Rittberg R, Dissanayake T, Katz SJ (2016). A qualitative analysis of methotrexate self-injection education videos on YouTube. Clin Rheumatol.

[15] Singh AG, Singh S, Singh PP (2012). YouTube for information on rheumatoid arthritis--a wakeup call?. J Rheumatol.

[16] Wasserman M, Baxter NN, Rosen B, Burnstein M, Halverson AL (2014). Systematic review of internet patient information on colorectal cancer surgery. Dis Colon Rectum.

[17] Drozd B, Couvillon E, Suarez A (2018). Medical YouTube videos and methods of evaluation: literature review. JMIR Med Educ.

[18] Nour MM, Nour MH, Tsatalou OM, Barrera A (2017). Schizophrenia on YouTube. Psychiatr Serv.

[19] Loeb S, Sengupta S, Butaney M (2019). Dissemination of misinformative and biased information about prostate cancer on YouTube. Eur Urol.

[20] Basch CH, Basch CE, Hillyer GC, Reeves R (2015). YouTube videos related to skin cancer: a missed opportunity for cancer prevention and control. JMIR Cancer.

[21] Okagbue HI, Ogutunde PE, Bishop SA (2020). Review on the reliability of medical contents on YouTube. iJOE.

[22] O'Connor C, Murphy M (2020). Going viral: doctors must tackle fake news in the covid-19 pandemic. BMJ.

[23] Adhikari J, Sharma P, Arjyal L, Uprety D (2016). YouTube as a source of information on cervical cancer. N Am J Med Sci.

[24] Cetin A (2021). Evaluation of YouTube video content related to the management of hypoglycemia. Cureus.

[25] Kunze KN, Cohn MR, Wakefield C (2019). YouTube as a source of information about the posterior cruciate ligament: a content-quality and reliability analysis. Arthrosc Sports Med Rehabil.

[26] Chapman D, Weaver A, Sheikh L, MacCormick AD, Poole G (2021). Evaluation of online videos of laparoscopic sleeve gastrectomy using the LAP-VEGaS guidelines. Obes Surg.

